# Eighty-nine and in Practice

**Published:** 1897-07

**Authors:** 


### EIGHTY-NINE AND IN PRACTICE.

At the recent meeting of the Susquehanna Dental Association at Carbondale, Pa., it was very gratifying to the writer to meet Dr. Otis Avery, of Honesdale, Pa., and listen to a paper of marked interest from his pen. This will be found upon another page of the journal, together with a very faithful representation of one of the oldest, if not *the* oldest man in practice in the United States. The reading of the essay, combined with the intellectual and physical force of Dr. Avery, produced a decided impression upon the meeting, demonstrating that, properly conducted, the practice of dentistry can be made conducive to long and vigorous life in the same degree as other and, supposed, more healthy occupations.

The Susquehanna Dental Association furnished a most excellent programme; indeed, it proved one of the most interesting meetings the writer has been privileged to attend in recent years, and socially the most enjoyable. This was largely due to the able Executive Committee and the large-hearted hospitality of the members and the dentists of the locality, who spared no efforts to make every one feel at home and enjoy the beautiful country of which Carbondale forms a very important part.

